# Protecting the fabric of society? Heterosexual views on the usefulness of the anti-gay laws in Barbados, Guyana and Trinidad and Tobago

**DOI:** 10.1080/13691058.2016.1207806

**Published:** 2016-07-22

**Authors:** Mahalia Jackman

**Affiliations:** ^a^ Cathie Marsh Institute for Social Research, School of Social Sciences, University of Manchester, Manchester, UK

**Keywords:** Homosexuality, attitudes, Caribbean, LGBT rights, anti-gay laws, perceived threat

## Abstract

This study evaluated the extent to which people living in Barbados, Guyana and Trinidad and Tobago believe that the anti-gay laws currently in place: (1) reflect moral standards; (2) stop the spread of homosexuality; (3) are important from a public health perspective; and (4) protect young people from abuse. Analysis reveals that demographics, religion, interpersonal contact and beliefs about the origin of homosexuality all influenced an individual’s views on the usefulness of the anti-gay laws in these states, but the significance of their impacts varied substantially across the arguments.

## Résumé

Cette étude a évalué dans quelle mesure les personnes qui vivent à la Barbade, au Guyana et à Trinidad et Tobago croient que les lois anti-gays actuellement en vigueur : (1) reflètent les normes morales ; (2) empêchent la propagation de l’homosexualité ; (3) ont leur importance pour une vision axée sur la santé publique ; et (4) protègent les jeunes des abus. L’analyse révèle que la démographie, la religion, les contacts interpersonnels et les croyances sur l’origine de l’homosexualité ont tous une influence sur les points de vue individuels sur l’utilité des lois anti-gays dans ces pays, mais que l’importance de leur impact varie considérablement d’un argument à l’autre.

## Resumen

En este estudio se evalúa en qué medida las personas que viven en Barbados, Guyana y Trinidad y Tobago creen que las leyes contra la homosexualidad actualmente en vigor: (1) reflejan los principios morales; (2) detienen la propagación de la homosexualidad; (3) son importantes desde una perspectiva de salud pública; y (4) protegen a los jóvenes contra abusos. El análisis indica que los datos demográficos, la religión, el contacto interpersonal y las creencias sobre el origen de la homosexualidad son factores que influyen en la percepción de las personas sobre la utilidad de las leyes contra la homosexualidad en estos Estados, pero el significado de los efectos que produce varía en gran medida según el argumento.

## Introduction

The rights of same-sex couples currently stand as one of the most contested issues across the globe. However, while discourse on gay rights in the West focuses on the marital and adoption rights of same-sex couples, in many countries across the globe, consensual adult same-sex intimate acts remain criminalised – including in Barbados, Guyana and Trinidad and Tobago (henceforth TT). In particular, these three states currently criminalise acts of buggery (anal sex), where the maximum penalties range from 25 years imprisonment in TT, to life imprisonment in Barbados and Guyana. These states also criminalise acts of ‘gross/serious indecency’ and, if convicted, persons may face up to two years imprisonment in Guyana, five years in TT and 10 years in Barbados – see Carroll and Itaborahy (2015) for details on the laws.

The anti-gay laws of Barbados, Guyana and TT were first imposed during British colonialism. In an attempt to import British morality to these states and protect British soldiers and colonial administrators from ‘corruption’, Britain subjected its colonies to various anti-sodomy provisions (Gupta and Long 2008; Han and O'Mahoney 2014). Interestingly, colonial thinking about homosexuality still appears alive and well today. Specifically, since gaining independence from the UK, Barbados, Guyana and TT have overhauled and revised many laws governing sex crimes (Robinson 2009), but have opted to keep pieces of legislation that signal heterosexuality as the ‘norm’.

Notwithstanding the obvious problems these antigay laws create for same-sex couples living in these three states, Jackman (2016) notes that, currently, the law is not normally used to police sexuality in practice. Thus, if consensual same-sex conduct is rarely penalised, why are these state adamant about keeping these laws on the books, especially since gay rights are increasingly being seen as important human rights? In this paper, I posit that the choice to keep the laws on the books may be due to the perceived threat of gay men and lesbians, and fear of what abolition of the laws may bring.

According to Stephan and Stephan’s (2000) integrated theory of intergroup threat, symbolic and/or realistic threats may act as causal antecedences of intergroup prejudice. Realistic threats pertain to perceptions that an out-group endangers the welfare of the in-group (inclusive of the in-group’s existence, political and/or economic power or physical wellbeing), while symbolic threats refer to the perception that the out-group threatens the in-group’s way of life, due to perceived group differences in values, standards, beliefs and morality (Brambilla and Butz 2013; Stephan and Stephan 2000). The anxiety elicited by these feelings of threat can lead members of the in-group to dislike members of the out-group. It is widely accepted that the greater the extent to which an in-group views the out-group as a threat (symbolic or realistic), the more negative the attitudes are likely to be (Vincent, Peterson, and Parrott 2009).

Gay men and lesbians are often perceived as sources of both symbolic and realistic threat to society. This perception is grounded in acceptance of negative stereotypes – mostly exaggerated and ill-founded beliefs – about gay men and lesbians (Herek 1991). For instance, gay men and lesbians are often stigmatised as immoral, violators of gender norms, child abusers and threats to public health (Bhugra 1987; Herek 1991, 2012; Wiley and Bottoms 2013). It is thus not surprising that one of the most cited arguments levied against extending basic human rights to gay men and lesbians is that such actions will harm the fabric of society (Bull, Pinto, and Wilson 1991; Hough 2004; Nichols 1984). It follows that if persons genuinely believe that gay men and lesbians or same-sex sexual acts harm society, then they would be more inclined to support anti-gay laws; they would not see it fit to extend basic human rights to individuals who they believe threaten their way of life. Thus, understanding the perceived usefulness of the anti-gay laws in Barbados, Guyana and TT is warranted, as it may aid advocates in understanding some of the roots of anti-gay sentiment in these states.

Against this backdrop, this study uses nationally representative data from the 2013 Caribbean Development Research Services (CADRES) Attitudes Towards Homosexuals survey to analyse the perceived usefulness of the anti-gay laws in Barbados, Guyana and TT. Specifically, I analyse subscriptions to notions that the anti-gay laws: (1) reflect moral standards; (2) stop the spread of homosexuality; (3) are important from a public health perspective; and (4) protect young people from abuse. This study has two aims. First, it seeks to determine the level of support for the above ‘anti-gay laws protect the fabric of society’ arguments. Second, it seeks to determine the extent to which popular correlates of anti-gay attitudes – that is, demographic factors, religion, interpersonal contact with gays and lesbians and beliefs about the origins of homosexuality – explain subscriptions to these arguments. The rest of this paper is organised as follows: the next section provides a brief review of the related literature, this is followed by a description of the data and empirical model, the penultimate section presents the results, and the study concludes with a discussion of the implications of the results and the limitations of the study.

## Review of the related literature

Currently, there is a well-established body of research on anti-gay bias in Western countries (mainly the USA), and the principal findings of this research have been summarised several times – see, for example, Bhugra (1987), Herek (1991) and Mason and Barr (2006). According to Mason and Barr (2006). Some of the most frequent findings in the West are that persons with negative views of gay men and lesbians are more likely to be religious, older, less highly educated and have had little to no interpersonal contact with gay men or lesbians. Mason and Barr also point out that the literature often finds that men tend to manifest higher levels of sexual prejudice than women – possibly due to the fact that men place heavier emphasis on traditional gender roles than women.

A subset of researchers have linked being married – an event associated with traditional lifestyles – to more conservative attitudes (Brumbaugh et al. 2008). Others have looked at variations in attitudes by ethnicity and race, though the findings on the impact of race can be best described as mixed – some studies find that Whites hold more negative attitudes, some find the reverse, while others find no significant racial variations (Herek and Capitanio 1995; Jenkins, Lambert, and Baker 2009; Negy and Eisenman 2005).

More recently, a few studies have focused on how beliefs about the origins of homosexuality influence attitudes towards gay men and lesbians. This research stems from attribution theory, which, in its most basic form, supposes that the perceived cause or controllability of behaviour influences how individuals view a stigmatised group or behaviour (Weiner 1979; Weiner, Perry, and Magnusson 1988). Research suggests that individuals who perceive homosexuality as innate tend to have more positive attitudes towards gay men and lesbians than those who perceive it as a choice (Haider-Markel and Joslyn 2008; Lewis 2009).

As alluded to earlier, the majority of research on anti-gay prejudice has been conducted using samples from the US. The extent to which these findings can be applied to the countries within the Commonwealth Caribbean^1^ (specifically, those under investigation in this study) has not been well established. Only a small body of work has been published on anti-gay bias in the Caribbean, most of which tends to be qualitative/ethnographic in nature (Gromer et al. 2013).

In general, these reports portray citizens in the Commonwealth Caribbean as intolerant of gay men and lesbians (Gromer et al. 2013). Given the antigay laws in the region, this claim seems plausible. However, Murray (2006, 2009) suggests that the claim that the region is homophobic is both correct and incorrect. Murray believes that Caribbean attitudes towards lesbians and gay men are complex, even in instances where sexual stigma and prejudice is prevalent there are inclusive/free spaces for gay men and lesbians.

With respect to the drivers of the anti-gay bias, researchers have speculated that adherence to traditional gender role ideologies is a major contributor to prejudice against gay men (Atluri 2001; Hope 2010; Linden Lewis 2003). In the Caribbean, sexual prowess with women is a key aspect of masculinity (Kempadoo 2009) and so men who have sex with men may challenge the cultural beliefs about what constitutes socially appropriate male behaviours (Maiorana et al. 2013). However, there are differences in the extent to which persons in these states adhere to traditional gender role ideologies. For example, qualitative research on gender in the Caribbean suggests that Barbadians are much more tolerant of non-traditional gender roles than their Jamaican neighbours (Marshall and Maynard 2009).

A more general theme in the published work on the Caribbean is that anti-gay prejudice is largely driven by conservative Christian beliefs (Douglas 2007; Gaskins 2013; Gutzmore 2004). In fact, the anti-gay laws in the Caribbean are often argued to be a reflection of their Christian beliefs (Abramschmitt 2008; Reding 2003). Another popular narrative is that negative stereotypes of gay men and lesbians lead to anti-gay sentiment. In the region, public discourse often conflates homosexuality with paedophilia and immorality, and also causally links homosexuality with HIV (Genrich and Brathwaite 2005; Gutzmore 2004; Rutledge and Abell 2005; White and Carr 2005). Since these are considered very serious threats to society, some individuals in the Caribbean may then believe that the harsh condemnations of gay men and lesbians are somewhat justified. In fact, some public officials in Barbados and TT have justified the anti-gay laws on the grounds of public morality and the need to protect the fabric of society. For instance, after UK Prime Minister, David Cameroon, stated in 2011 that the UK Government would possibly cut or withhold foreign aid to countries with anti-gay laws on the books (BBC 2011), the then Minister of Youth, Family and Sport in Barbados, Stephen Lashley, noted:

The question is: are we going to surrender our tried and tested values for all these things that are now becoming the flavour of the international community? I say No. (Marshall 2011)

Meanwhile, in 2007, religious leaders in TT unsuccessfully tried to convince the House of Assembly to bar Elton John – an openly gay singer and musician – from performing in Tobago’s national jazz festival for fear that he would encourage others to join his lifestyle (Daily Mail 2007).

Interestingly, very few studies have emerged to quantitatively investigate these attitudes and behaviours. In fact, many of the quantitative studies that do exist are sampled predominantly from university bodies. Gromer et al. (2013), for instance, studied attitudes towards gay men using a convenience sample of university students in Barbados. Gromer et al. did not find a relationship between contact with gay men or lesbians and sexual prejudice. But they did find that: (1) men had more negative attitudes towards gay men, but there was no evidence of gender gap with respect to attitudes towards lesbians; and (2) higher levels of religiosity was associated with greater prejudice to both gay men and lesbians. Chadee et al. (2013) studied the attitudes of 204 university students in TT and found that the highly religious were the most likely to be intolerant. Meanwhile, West and Hewstone (2012) investigated the relationship between contact with gay men and attitudes towards gay men using a sample of Jamaican students, and compared it to the corresponding relationship for British students. West and Hewston found that Jamaican students reported more negative attitudes than their British equivalents , but that contact was more strongly associated with a reduction in anti-gay prejudice for the Jamaican sample than for Britons. However, the generalisability of studies using university students is questionable. University students tend to be younger and better educated than the national population, making it difficult to use these studies to draw inferences about the attitudes of the mass public.

Recently, Jackman (2016) and West and Cowell (2015) have attempted to remedy this problem. Employing nationally representative data attained from CADRES, Jackman investigated general support for the maintenance and enforcement of the anti-gay laws in Barbados, Guyana and TT. The results suggest that the individual most likely to support both the retention and enforcement of the anti-gay laws is strongly religious, does not identify as a White Caribbean, is in a common-law marriage, resides in TT, has no gay friends and believes homosexuality is a choice. In contrast to the aforementioned literature on the West, neither age nor education predicted attitudes towards law retention.

West and Cowell’s (2015) study was specific to Jamaica. Using nationally representative survey data, they investigated the impact of age, gender, education, income, religiosity and music choice (dancehall)^2^ on four measures of anti-gay prejudice: (1) negative attitudes; (2) social distance; (3) opposition to gay rights; and (4) negative behaviours. Only gender, religiosity and music choice were significantly related to all four measures of anti-gay prejudice. Men and those with a preference for dancehall were more likely to express anti-gay prejudice than women or persons preferring other genres of music. Religiosity, on the other hand, was associated with more negative attitudes, greater social distance and greater opposition to gay rights, but was also associated with less negative behaviours towards gays and lesbians. Age did not predict the social distance or attitudinal measures. However, older persons did report greater opposition to gay rights, but were also less likely to report engaging in negative behaviours. Finally, participants with higher levels of education and income generally reported less negative attitudes, social distance and negative behaviours, but these two variables had no impact on opposition towards gay rights.

While work by Jackman (2016) and West and Cowell (2015) shed some light on anti-gay bias in the Commonwealth Caribbean, neither of these studies looked at the extent to which disapproval could possibly stem from negative stereotypes that portray gays and lesbians as a threat to society. This study adds to the current body of literature by evaluating the extent to which persons in Barbados, Guyana and TT view the anti-gay laws as ‘protective’ measures. It will also empirically investigate the factors influencing such beliefs. As alluded to earlier, many of the studies on the West have found that demographic variables, religion, interpersonal contact and beliefs about the origins of homosexuality are among the chief determinants of anti-gay prejudice. In what follows, I will investigate whether these social and psychological factors also impact the likelihood than an individual residing in Barbados, Guyana and TT will view the anti-gay laws as useful.

## Data and measures

### Estimation sample

This study uses secondary data from the 2013 CADRES Attitudes Towards Homosexuality survey, which was conducted in three Caribbean states – Barbados, Guyana and TT. Details about the survey can be found in the CADRES reports (Caribbean Development Research Services 2013a, 2013b, 2013c). A total of 2871 adults (age 18 and over) were surveyed: 830 Barbadians, 1034 Guyanese and 1007 persons from TT. This study focuses on the responses of those individuals who listed their sexual orientation as heterosexual. Once missing observations were removed from both the dependent and independent variables, the estimation sample consisted of 2085 individuals. Throughout this paper, I focus on results from the estimation sample.

### Measures

#### Dependent variables

Respondents were asked about their beliefs regarding the importance of the current buggery/sodomy laws. Specifically, respondents were asked:

Based on your understanding of the (insert country here) laws with respect to buggery/sodomy, which of these objectives do you think that the laws achieve in their present form?

(1) The laws are a fair and reasonable expression of our moral standards. (Yes, No, Unsure/won’t say)
(2) The laws help to stop the “spread” of homosexuality. (Yes, No, Unsure/won’t say)
(3) The laws are important from a Public Health perspective. (Yes, No, Unsure/won’t say)
(4) The laws protect young people from abuse. (Yes, No, Unsure/won’t say)

These four dependent variables are first dichotomised such that an item takes on a value of 1 if the respondent’s answer to a questionnaire item was ‘Yes’ and 0 if the respondent chose ‘No’ or ‘Unsure/Won’t say’. In this way, the study is focused on modelling the likelihood of a person believing that the laws are useful in some form.

#### Predictors

Table 1 provides some descriptive statistics for the predictors, and the presentation of most variables is largely a reflection of the response options provided by CADRES. All variables are categorical. The demographic variables in the study are age, gender, race, marital status, level of education (split into those that attended a tertiary institution and those that did not)^3^ and country of residence. Three of the survey’s items can be used to test the impact of religion. Respondents were asked about their religious identities, religious participation (whether they consider themselves active, passive or were unsure/prefer not to say) and their source of views on sexuality. The interpersonal contact variable used in this paper was constructed using two survey items. First, the CADRES survey asked if the respondents had any gay friends and any gay family members and for both questions respondents were given the response choices ‘Yes’, ‘No’ or ‘Prefer not to say’. Responses were then recoded to create a four-category variable, such that: (1) if a person did not state having friends or family members who identified as a gay man or lesbian, they were denoted as having no close gay associates; (2) if they chose ‘yes’ to only one category (either yes to having gay friends or having a gay family member), they were denoted as having at least one close gay association; (3) if they chose ‘yes’ for both items, they were denoted as having both friends and family that identified as gay; and (4) if the individual chose ‘Unsure/prefer not to say’ for both categories, or a combination of ‘No’ and ‘Unsure/prefer not to say’, they were denoted as having an undetermined number of associations. Finally, beliefs about the origins of homosexuality were captured via a 6-item variable presented as: (1) Birth defect; (2) Psychological trauma/sexual abuse; (3) Lack of/poor moral or religious groundings/bad parenting; (4) Choose to be that way; (5) Born that way; and (6) Unsure/prefer not to say.

**Table 1.** Descriptive statistics.

Variable	(%)	Variable	(%)
*Age*		*Religious identity*	
18–30	35.68	Evangelical Christian	39.71
31–50	34.00	Non-evangelical Christian	26.95
51 and over	30.31	Muslim	4.89
		Hindu	11.27
*Gender*		Other	10.65
Male	49.78	Not religious	2.83
Female	50.22	Did not state	3.69
			
*Marital status*		*Religious participation*	
Single	44.65	Active	48.49
Married	34.53	Passive	42.64
Married (common law)	6.57	Unsure/won’t say	8.87
Other	14.24		
		*Source of views on human sexuality*	
*Race*		Non-religious sources	40.67
Black	54.59	Religion	48.30
White	1.29	Unsure/prefer not to say	11.03
Indo Caribbean	20.38		
Mixed	18.66	*Contact* – *friends and family*	
Other	4.08	No close gay associations	51.51
		At least one	29.69
*Education*		Both friends and family	12.76
No tertiary	73.05	Undetermined number of associations	6.04
Tertiary	26.95		
		*Attribution*	
*Country*		Birth defect	5.28
Barbados	28.73	Psychological trauma/sexual abuse	14.44
Guyana	29.59	Lack of/poor moral or religious grounding/bad parenting	14.87
Trinidad and Tobago	41.68	Chose to be that way	34.82
		Born that way	17.03
		Unsure/prefer not to say	13.57

Note: Based on the estimation sample: 2085 observations.

### Statistical analysis

The dependent variables were dichotomised for the empirical analysis. A look at the tetrachoric correlations^4^ shown in Table 2 suggests that these four dependent variables are positively correlated. It is quite possible that this correlation may continue to exist even after regressing the binary indicators on the aforementioned predictors. If true, then this interrelatedness would manifest itself via correlated error terms, and so estimating individual probit or logit models would yield inefficient estimates.

**Table 2.** Tetrachoric correlations – dependent variables.

	Laws reflect morals	Laws stop the spread of homosexuality	Laws are important from a public health perspective	Laws protect young people from abuse
Laws reflect morals	1.00	0.55***	0.60***	0.66***
Laws stop the spread of homosexuality		1.00	0.71***	0.78***
Laws are important from a public health perspective			1.00	0.64***
Laws protect young people from abuse				1.00

Note:

^***^ indicates statistical significance at the 0.1% level.

To deal with this problem, I opted to employ multivariate probit regressions. The term multivariate in this case refers to the fact that there are four dependent variables, and not the number of predictors. The multivariate probit model estimates the dependent variables jointly. It thus allows us to evaluate how the predictors impact the probability that one agrees with one of the arguments (say, laws reflect morals), while at the same time controlling for the fact that that argument is positively correlated with the other three arguments.

## Results

**Figure 1.**
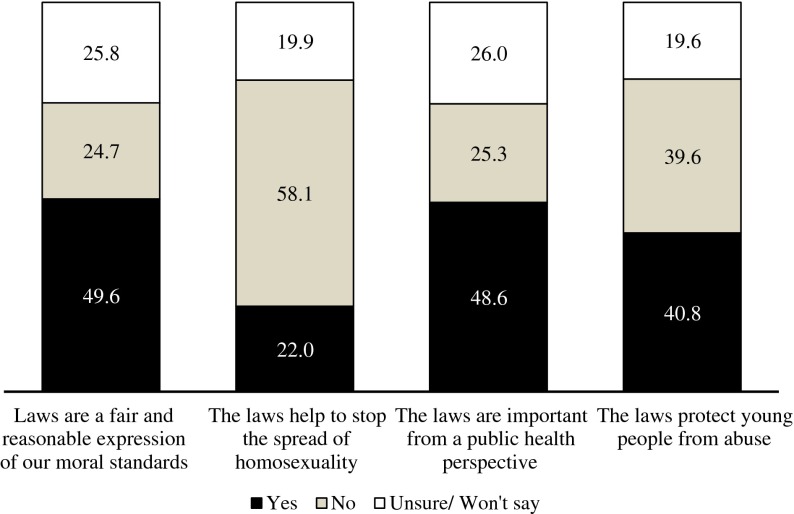
Beliefs about the usefulness of the laws (%).

The first aim of this study was to determine the prevalence of the view that the current antigay laws protect the fabric of society. Figure 1 shows the distribution of the dependent variables. Based on the estimation sample, 49.6% of heterosexual respondents believed the laws are in line with moral standards, 48.6% thought the laws are important from a public health perspective and 40.8% believed that the laws protect young people from abuse. The argument that the ‘Laws help to stop the spread of homosexuality’ had the least adherents, with 22.0% of respondents agreeing with this statement. A large portion of the sample (19–26%) were either unsure or did not want to state their opinions about the usefulness of the laws.

**Figure 2.**
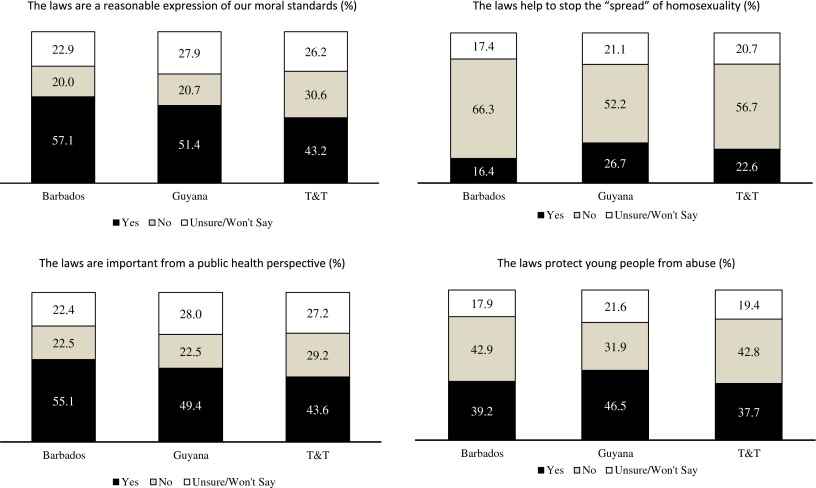
Beliefs about the usefulness of the laws banning anal sex (by country).

Figure 2 takes the analysis a step further and looks at support across the three countries. In line with the aggregated data, in each country, the ‘Laws are a fair and reasonable expression of our morals’ statement had the largest number of supporters, followed by the notion that the laws protect public health. The ‘Laws stop the spread of homosexuality’ argument has the least support. Figure 2 also suggests that the intensity of anti-gay sentiment differed across the countries. For instance, Barbados had the largest share of respondents agreeing that the laws were an expression of moral standards. Barbados also had the largest share of persons agreeing that the laws were important from a public health perspective. Meanwhile, respondents from Guyana appeared the most likely to believe that the laws stop the spread of homosexuality and protect children from abuse.

Taken together, the analysis thus far indicates that a significant share of the sample believe that the anti-gay laws protect the fabric of their society. This raises the question: Who are the individuals most likely to conform to such notions? While at face value it seems that TT residents are less likely than their Barbadian and Guyanese neighbours to view the laws as protectionist measures, there are factors beyond country of origin that may influence subscribing to these arguments. As such, the dependent variables were dichotomised and estimated as a function of demographics, religion, interpersonal contact and beliefs about the origin of homosexuality, via a multivariate probit model. The empirical model was estimated with all predictors in the model at the same time, so that one can assess the impact of a variable, *ceteris paribus* ^5^. Table 3 presents the results. The 4-equation model is characterised by six cross-equation correlation terms, *ρ*_12_, *ρ*_13_, *ρ*_14_, *ρ*_23_, *ρ*_24_ and *ρ*_34_, which represent the correlations among each of the dependent variables that exist, even after controlling for the various predictors. As shown in the Table, each cross-correlation was statistically significant, confirming that the system estimation is more appropriate than individual probit/logit regressions.

**Table 3.** Perceptions of the usefulness of the anti-gay laws.

	Laws reflect morals (1)		Laws stop the spread of homosexuality (2)		Laws are important from a public health perspective (3)		Laws protect young people from abuse (4)	
	Prob.	χ^2^	Prob.	χ^2^	Prob.	χ^2^	Prob.	χ^2^
*Age*								
18–30	0.51	1.39	**0.20**	6.46^*^	0.48	0.59	0.40	0.17
31–50	0.48		**0.25**		0.48		0.40	
51 and over	0.49		**0.22**		0.50		0.41	
								
*Gender*								
Male	0.49	0.35	**0.26**	3.88^*^	0.48	0.14	0.41	0.24
Female	0.49		**0.21**		0.49		0.40	
								
*Education*								
No tertiary	0.50	0.49	**0.23**	3.87^*^	0.49	0.15	0.40	0.22
Tertiary	0.48		**0.19**		0.48		0.42	
								
*Marital status*								
Single	0.48	4.06	0.22	4.01	0.48	1.19	0.41	2.79
Married	0.49		0.20		0.50		0.43	
Common law	0.54		0.19		0.47		0.40	
Other	0.53		0.21		0.49		0.42	
								
*Race*								
Black	**0.47**	13.45^**^	**0.20**	9.56^*^	0.49	3.21	**0.39**	14.23^**^
White	**0.49**		**0.18**		0.54		**0.21**	
Indo	**0.59**		**0.30**		0.52		**0.49**	
Mixed	**0.47**		**0.20**		0.46		**0.37**	
Other	**0.48**		**0.20**		0.44		**0.39**	
								
*Country*								
Barbados	**0.61**	50.29^***^	**0.19**	11.74^**^	**0.56**	26.61^***^	**0.42**	10.35^**^
Guyana	**0.49**		**0.28**		**0.51**		**0.45**	
Trinidad and Tobago	**0.42**		**0.20**		**0.42**		**0.36**	
								
*Religious identity*								
Evangelical	0.50	10.46	0.21	4.17	0.48	6.04	0.43	6.30
Non-evangelical Christian	0.49		0.22		0.49		0.38	
Muslim	0.60		0.27		0.44		0.41	
Hindu	0.47		0.21		0.49		0.41	
Other	0.51		0.23		0.51		0.37	
Not religious	0.40		0.19		0.36		0.34	
No response	0.39		0.29		0.51		0.47	
								
*Religious participation*								
Active	**0.51**	7.39^*^	0.24	3.80	**0.52**	12.52^**^	**0.43**	10.97^**^
Passive	**0.50**		0.21		**0.46**		**0.40**	
Unsure/won’t say	**0.40**		0.18		**0.39**		**0.30**	
								
*Views on sexuality*								
Non-religious source	**0.46**	19.74^***^	0.21	0.87	0.49	0.71	**0.39**	**5.19**^**+**^
Religion	**0.54**		0.23		0.48		**0.43**	
Unsure/prefer not to say	**0.40**		0.23		0.46		**0.36**	
								
*Contact* – *friends and family*								
No associations	**0.51**	7.86^*^	**0.25**	12.40^**^	**0.52**	21.66^***^	0.42	2.85
At least one	**0.50**		**0.21**		**0.48**		0.41	
Both friends and family	**0.48**		**0.19**		**0.46**		0.39	
Undetermined number of associations	**0.38**		**0.14**		**0.32**		0.35	
								
*Origin of homosexuality*								
Birth defect	**0.47**	19.40^**^	**0.13**	21.69^***^	**0.44**	24.51^***^	**0.35**	13.10^*^
Psychological trauma/sexual abuse	**0.57**		**0.28**		**0.59**		**0.49**	
Parenting, poor morals, etc.	**0.50**		**0.26**		**0.51**		**0.37**	
Chose to be that way	**0.51**		**0.23**		**0.49**		**0.41**	
Born that way	**0.47**		**0.18**		**0.43**		**0.39**	
Unsure/prefer not to say	**0.40**		**0.19**		**0.43**		**0.38**	
								
Correlated errors:								
*ρ*_12_	**0.56**	(0.03)^***^						
*ρ*_13_	**0.59**	(0.03)^***^						
*ρ*_14_	**0.66**	(0.02)^***^						
*ρ*_23_	**0.71**	(0.03)^***^						
*ρ*_24_	**0.78**	(0.02)^***^						
*ρ*_34_	**0.64**	(0.02)^***^						

Notes:

^***^ indicates statistical significance at the 0.1% level.

^**^ indicates statistical significance at the 1% level.

^*^ indicates statistical significance at the 5% level.

^+^ indicates statistical significance at the 10% level.

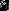
 indicates the correlation between the errors between equation *i* and equation *j*.

Prob. means predicted probability. Figures in bold denote variables that were found to be statistically significant.

A general consensus in the literature is that interpretation of the coefficients in these models is not straightforward (Cameron and Trivedi 2010). As such, I use predicted probabilities, which are much easier to interpret. In this paper, the predicted probabilities give an estimate of the likelihood that an individual with a specific characteristic (say in a particular age group) agrees with a particular fabric of society argument. The significance of each predictor was tested via a chi-square (χ^2^) test, where the null hypothesis was that the predicted probabilities are all equal. For quick reference, the variables that were found to be statistically significant are shown in bold.

Looking first at demographic factors, the findings suggest that most demographic variables do not consistently predict heterosexual beliefs about the usefulness of the anti-gay laws. For instance, the marital status variable was insignificant across the board, while the age, gender and education variables were only significant in the ‘Laws stop the spread of homosexuality’ equation. Women, persons under 30 and tertiary educated individuals seem to be the least likely to think the laws affect the spread of homosexuality. Race mattered for three of the four arguments: Indo-Caribbeans were the most likely to view the laws as a means of upholding moral standards, stopping the spread of homosexuality and protecting children from abuse. The only demographic variable that was significant in each equation was country of residence. In line with what was shown in Figure 2, residents of Barbados were the most likely to agree that the laws are an expression of moral standards and that the laws are important from a public health perspective. Respondents from Guyana had the highest probability of stating that the laws stop the spread of homosexuality and that they protect children from abuse.

Turning now to the impact of religion, there is no evidence that opinions varied substantially across religious identities. Rather, religious participation, and whether or not one’s views on sexuality were shaped by religion, seem more important. Compared to those who identified as passively involved in their religion, those who identified as active were more likely to view the laws as a means of protecting moral standards, public health and children from abuse. Meanwhile, individuals whose views on sexuality were shaped by religion had the greatest likelihood of supporting the anti-gay laws from a moral perspective and/or to limit child abuse. As a side note, none of the religious variables had a significant impact on the argument relating to the spread of homosexuality.

Subscriptions to the view that the anti-gay laws are useful from moral and health perspectives, or that they stop the spread of homosexuality, varied with the amount of contact with gay men and lesbians. In each of these three equations, those who stated they have no close contact with lesbians or gay men consistently had the highest predicted probabilities of agreeing with the anti-gay statements. The models also imply that those with more channels of contact (both family and friends that identify as gay) had a slightly smaller probability of viewing gays and lesbians as a threat, relative to those with only one contact – hinting that the number of interactions may matter.

Finally, beliefs about the origin of homosexuality were significant in each equation. Persons who believed that homosexuality was caused by some traumatic event were the most likely to view the anti-gay laws as necessities, followed closely by people who stated that homosexuality was a choice, or believed that it was due to bad upbringing or poor morals.

## Discussion

Using large-sample nationally representative surveys, this paper has evaluated the extent to which citizens of Barbados, Guyana and TT believe that the anti-gay laws protect their society from harm. The results imply that public support for these laws is grounded in negative stereotypes about gay men and lesbians. It logically follows that a key avenue for changing anti-gay attitudes in these states lies in breaking strongly rooted stereotypes about gay men and lesbians.

The results suggest that subscriptions to ‘Homosexuality harms the fabric of society’ arguments are largely related to a persons’ beliefs about the origins of homosexuality. Of particular interest, persons who believed that homosexuality was innate (either by simply stating that persons were born gay or believed that homosexuality was a ‘birth defect’) or were unsure about the origins of homosexuality, appeared least likely to agree that the antigay laws protect the society from harm. However, persons who believed that homosexuality was caused by trauma, bad parenting or was a choice were consistently the most likely to assume the laws somehow protected the society from harm. The main implication is that widespread changes in views on the origin of homosexuality could reduce negative stereotypes of gay men and lesbians.

An interesting observation was that persons attending university and younger generations still held on to the view that gay men and lesbians were immoral, spread diseases and abuse children, and so believed that the anti-gay laws are a necessity. Age and education only mattered for responses to the statement that the laws prevent the spread of homosexuality. With younger and educated persons still holding onto the more severe negative stereotypes of gay men and lesbians, it seems that cohort replacement and the current trend of increasing education levels in these states may not lead to a substantial decline in anti-gay bias, at least not in the near future.

With respect to the impact of religion, there was no evidence to suggest that views on the usefulness of the laws varied across religious identities. However, religious participation and whether or not one’s views on sexuality were shaped by religion mattered. Specifically, in this sample, individuals who identified as being active in their religion were more likely to think that same-sex behaviours needed to be admonished to protect society’s morals, stop the spread of homosexuality and protect young children from abuse than those who identified as passive in their religion. Persons whose views on sexuality came from religious sources were also more likely to believe that the laws are important from a moral perspective and were more likely to think the laws protect young persons from abuse. These findings are indeed plausible. Durkheimian theories of social integration suggest that the more one is integrated into a social group, the more likely one is to conform to its norms (Scheepers, Grotenhuis, and Slik 2002). Given that several religious doctrines characterise same-sex intimate acts as sinful, it seems logical that those with greater religious exposure, or for whom religion is more salient, would be more likely to view homosexuality in a negative light.

Finally, there is evidence that greater interaction with lesbians and gay men could aid in de-stigmatising them. Taken at face value, it would seem as though greater visibility of lesbians and gay men could be a channel for more positive attitudes towards homosexuals. However, in these states, homosexuality is very much stigmatised and so persons may be less likely to come out as lesbian or gay. Thus, to encourage visibility and increase social contact, legislative efforts to curtail anti-gay bullying would be needed.

Taken together, this study has showcased the extent to which individuals living in Barbados, Guyana and TT support the antigay laws due to the perceived threat of gay men and lesbians. A major strength of the study was the use of nationally representative survey data and the fact that it focused on a sample of Commonwealth Caribbean states – a region often critiqued for its high levels of antigay sentiment, but for which few national-level empirical studies exist. However, the study is not without its limitations. For instance, the perceived threat variable was not gender-specific. An area of future work could therefore be to explore whether the perceived threats differ for gay men and lesbians. Another limitation relates to the large number of respondents who selected ‘Unsure/won’t say’ when asked about their views on the usefulness of the anti-gay laws. In general, the lack of definitive responses to the above items could be reflective of a social desirability bias. While participants in the CADRES surveys were given the option to complete the survey themselves, the primary mode of data collection was via face-to-face interviews. In a context where homosexuality is stigmatised, it is possible that some participants chose the ‘Unsure/won’t say’ option for these items in order to avoid giving socially undesirable responses. The lack of definitive responses could also be due to the survey measure used. As noted by Lamarca (2011), attitudes, beliefs and opinions in a population generally exist on a vast multidimensional continuum. Hence, some respondents may not have been able to give a concrete ‘Yes’ or ‘No’ answer on this issue and may have, by default, opted for ‘Unsure/won’t say’. Perhaps a Likert scale would have been more appropriate. Finally, 11 of the 12 Commonwealth Caribbean states have laws penalising consensual same-sex intimacy, as The Bahamas decriminalised ‘buggery’ and lesbian practices in 1991. However, this study only addressed three of those states due to constraints on data availability. The research could have benefited from a larger sample of Commonwealth Caribbean states.

With these limitations, this study serves as a first step into evaluating attitudes towards bans on sexual practices in the Commonwealth Caribbean. Further research needs to be undertaken to complement this research and to possibly address some of the limitations of this study. Indeed, the more we know about antigay bias in these states, the more likely it is that efficacious programmes and strategies can be developed and implemented to persuade individuals to reject anti-gay prejudice.

## Disclosure statement

No potential conflict of interest was reported by the author.

## Funding

This work was supported by the Economic and Social Research Council [grant number ES/J500094/1].

## Notes

1. The Commonwealth Caribbean states are the former British colonies: Antigua and Barbuda, The Bahamas, Barbados, Belize, Dominica, Grenada, Guyana, Jamaica, St Kitts and Nevis, St Lucia, St Vincent and the Grenadines, and Trinidad and Tobago.

2. Music choice was whether or not the participant had a preference for dancehall. Dancehall is a style of music indigenous to Jamaica. As noted by West and Cowell, some of the music contains harsh condemnations of homosexuality.

3. Education was initially a 5-category variable and was dichotomised by the author. It should be noted that this recoding of the variable had no impact on the results, as there were no statistical differences in the attitudes of persons who were reported lower levels of education. The results available from author on request.

4. Correlation coefficients for binary variables.

5. As pointed out by one of the referees, it is possible that the impact of the demographics and religion variables could be mediated by contact and attribution variables. Hence, the model was first estimated without the contact or attribution variables, then with them. The statistical significance of the demographics and religion variables did not change upon adding the contact and attribution variables. The results of this analysis are available from author upon request.
